# MET-receptor targeted fluorescent imaging and spectroscopy to detect multifocal papillary thyroid cancer

**DOI:** 10.1007/s00259-023-06525-5

**Published:** 2023-11-29

**Authors:** Madelon J. H. Metman, Pascal K. C. Jonker, Luc H. J. Sondorp, Bettien M. van Hemel, Mark S. Sywak, Anthony J. Gill, Liesbeth Jansen, Paul J. van Diest, Tessa M. van Ginhoven, Clemens W. G. M. Löwik, Anh H. Nguyen, Dominic J. Robinson, Gooitzen M. van Dam, Thera P. Links, Rob P. Coppes, Rudolf S. N. Fehrmann, Schelto Kruijff

**Affiliations:** 1 Department of Surgery, University Medical Center Groningen, University of Groningen, Hanzeplein 1, 9713 GZ Groningen, the Netherlands; 2 Department of Endocrine Surgery and Surgical Oncology, Royal North Shore Hospital, St Leonards, Australia; 3 Department of Biomedical Sciences of Cell & Systems – Section Molecular Cell Biology, University Medical Center Groningen, University of Groningen, Groningen, the Netherlands; 4 Department of Pathology, University Medical Center Groningen, University of Groningen, Groningen, the Netherlands; 5 NSW Health Pathology, Department of Anatomical Pathology, Royal North Shore Hospital, St Leonards, Australia; 6 Sydney Medical School, University of Sydney, Sydney, Australia; 7 Cancer Diagnosis and Pathology Group Kolling Institute of Medical Research, Royal North Shore Hospital, St Leonards, Australia; 8 Department of Pathology, University Medical Center Utrecht, Utrecht, the Netherlands; 9 Sidney Kimmel Comprehensive Cancer Center, Johns Hopkins, Baltimore, USA; 10 Department of Surgery, Erasmus MC Cancer Institute, Rotterdam, the Netherlands; 11 Department of Radiology and Nuclear Medicine, Erasmus MC, Rotterdam, the Netherlands; 12 Department of Pathology, Erasmus MC, Rotterdam, the Netherlands; 13 Department of Otorhinolaryngology and Head and Neck Surgery, Erasmus MC Cancer Institute, Rotterdam, the Netherlands; 14 Department of Nuclear Medicine and Molecular Imaging, University Medical Center Groningen, University of Groningen, Groningen, the Netherlands; 15 AxelaRx/TRACER B.V, Groningen, the Netherlands; 16 Department of Endocrinology, University Medical Center Groningen, University of Groningen, Groningen, the Netherlands; 17 Department of Radiation Oncology, University Medical Center Groningen, University of Groningen, Groningen, the Netherlands; 18 Department of Medical Oncology, University Medical Center Groningen, University of Groningen, Groningen, the Netherlands

**Keywords:** Papillary thyroid cancer, Multifocality, Molecular fluorescence–guided imaging, Spectroscopy

## Abstract

**Purpose:**

Multifocal disease in PTC is associated with an increased recurrence rate. Multifocal disease (MD) is underdiagnosed with the current gold standard of pre-operative ultrasound staging. Here, we evaluate the use of EMI-137 targeted molecular fluorescence-guided imaging (MFGI) and spectroscopy as a tool for the intra-operative detection of uni- and multifocal papillary thyroid cancer (PTC) aiming to improve disease staging and treatment selection.

**Methods:**

A phase-1 study (NCT03470259) with EMI-137 was conducted to evaluate the possibility of detecting PTC using MFGI and quantitative fiber-optic spectroscopy.

**Results:**

Fourteen patients underwent hemi- or total thyroidectomy (TTX) after administration of 0.09 mg/kg (*n* = 1), 0.13 mg/kg (*n* = 8), or 0.18 mg/kg (*n* = 5) EMI-137. Both MFGI and spectroscopy could differentiate PTC from healthy thyroid tissue after administration of EMI-137, which binds selectively to MET in PTC. 0.13 mg/kg was the lowest dosage EMI-137 that allowed for differentiation between PTC and healthy thyroid tissue. The smallest PTC focus detected by MFGI was 1.4 mm. MFGI restaged 80% of patients from unifocal to multifocal PTC compared to ultrasound.

**Conclusion:**

EMI-137-guided MFGI and spectroscopy can be used to detect multifocal PTC. This may improve disease staging and treatment selection between hemi- and total thyroidectomy by better differentiation between unifocal and multifocal disease.

**Trial registration:**

NCT03470259

**Supplementary Information:**

The online version contains supplementary material available at 10.1007/s00259-023-06525-5.

## Introduction

The incidence of thyroid cancer has increased worldwide over the last decades. The most common thyroid cancer subtype is papillary thyroid cancer (PTC). PTC management traditionally consists of a total thyroidectomy, nodal dissection in case of lymph node metastasis, and adjuvant radioactive iodine treatment (RAI) [1]. Following treatment, patients with PTC have a 10-year disease-specific survival of up to 97% [2]. Despite the excellent prognosis, quality of life of patients with PTC is reduced by treatment-related morbidity [3–5]. Therefore, today’s PTC management aims to maintain the excellent survival rate and reduce disease recurrence but increasingly focuses on minimizing treatment-related morbidity and overtreatment. The 2015 American Thyroid Association (ATA) guidelines proposed a treatment de-escalation strategy towards hemithyroidectomy for patients with low-risk, 0–4 cm unifocal PTC, absence of extrathyroidal extension, and without nodal metastasis [1]. This paradigm shift was based on studies reporting similar survival and reduced morbidity following hemithyroidectomy in patients with low-risk PTC [6, 7]. However, the case mix of PTC tumors in other countries may be more aggressive as a result of less diagnostic imaging of the thyroid [8]. Therefore, implementing the 2015 ATA guidelines in these countries with different protocols may lead to undertreatment. This requires accurate pre-operative staging for high-risk tumor features such as multifocal disease [8]. Patients with multifocal disease are primarily treated with total thyroidectomy and RAI due to the increased risk of recurrence [1]. Ultrasound is the current gold standard for pre-operative staging to detect multifocality. Still, it fails to detect multifocal disease in 52% of patients during pre-operative staging, even in high volume, high expertise centers [9]. The reported positive and negative predictive values of ultrasound for detecting multifocal disease in differentiated thyroid cancer range from 44.4 to 57% and 63%, respectively [10, 11]. Endocrine surgeons need a more accurate imaging modality to identify patients with multifocal disease to prevent undertreatment and select patients who may benefit from total thyroidectomy despite the treatment-related morbidity.

Molecular fluorescence–guided imaging (MFGI) and quantitative multidiameter single-fiber reflectance and single-fiber fluorescence (MDSFR/SFF) spectroscopy of near-infrared fluorescent (NIRF) tracers are safe tools for in vivo tumor detection. These tools may be useful to rule out or detect multifocal disease in the ipsi- or contralateral thyroid lobe, thereby improving clinical decision-making. A recent study showed that the clinically available fluorescent-labeled tracer EMI-137 could identify true negative nodal compartments in patients with PTC using MFGI and spectroscopy reducing the number of negative prophylactic central compartment dissections by 25% [12]. The small peptide tracer targets the tyrosine-kinase receptor MET, localized in the plasma membrane. MET is encoded by the proto-oncogene *MET* and activated by its ligand, the hepatocyte growth factor (HGF) [13, 14]. MET is overexpressed in PTC and associated with increased extrathyroidal extension rates, higher tumor stages, more frequent nodal metastases, a higher prevalence of BRAF^V600E^ mutations, and an increased 10-year locoregional recurrence rate [12, 15–19]. In the present study, we aimed to assess if MFGI and MDSFR/SFF spectroscopy can detect primary tumor tissue and rule out multifocal disease after intravenous injection of EMI-137. We conducted a phase-1 study to evaluate the usage of EMI-137 targeted molecular fluorescence–guided imaging to detect PTC foci compared to healthy thyroid tissue. We calculated diagnostic accuracy of EMI-137 and compared MFGI-detected PTC foci to pre-operative ultrasound results. In addition, we quantified intrinsic fluorescence values of tumor tissue and healthy thyroid tissue using MDSFR/SFF. We used a multi-step analytical framework to demonstrate PTC tumor-specific targeting of EMI-137 on macroscopic and microscopic levels.

## Methods

### Study design

The study design has been previously outlined [12]. In short, a multicenter, phase-1 dose-escalation study was conducted on EMI-137 (Edinburgh Molecular Imaging Ltd, Edinburgh, UK), a fluorescent-labeled peptide targeting the MET receptor. From June 2018 to December 2019, this study aimed to determine the safety, feasibility, and ideal dosage for identifying PTC nodal metastases using the tracer. During this study, fluorescence data of the primary tumor and other tumor PTC foci were obtained from all included patients undergoing (hemi)thyroidectomy. The research received approval from local ethical boards, adhered to the Declaration of Helsinki (2013 Fortaleza, Brazil amendment), and took place at the University Medical Center Groningen and the Erasmus Medical Center. Patients older than 18 years, who had a pre-operative confirmation of PTC (Bethesda VI) through fine-needle aspiration and scheduled to undergo a (hemi)thyroidectomy with lymph node dissection for primary or recurrent disease, were eligible for inclusion in PTC nodal metastases study. From this cohort, individuals were selected for the current study. Written and signed informed consent was obtained. Considering the estimated half-life of 2 h and 30 min for the tracer, participants received an IV injection of EMI-137 (at doses of 0.09 mg/kg, 0.13 mg/kg, or 0.18 mg/kg) 2 h prior to their surgical procedure. The vital signs of patients were monitored for an hour after injection. Any adverse events were documented using the National Cancer Institute Common Terminology Criteria for Adverse Events (CTCAE) version 5.0. Serious adverse events were classified as grade 3 or higher.

### Ex vivo MFGI and quantitative spectroscopy

Back-table MFGI of the thyroid specimen was performed with an IVIS Spectrum imaging system and the IVIS Lumina II imaging system as an alternative system (PerkinElmer, Waltham, USA). Epi-fluorescence images with the IVIS Spectrum were taken using excitation/emission filter settings of 640/680 nm, an exposure of two seconds, and binning eight. For the IVIS Lumina II, excitation/emission filter settings of 640/595–770, a two-second exposure, and binning of four were used to acquire images of the tissue. Living Image (version 4.3.1) was used to draw regions of interest (ROI) on IVIS images. The size of all tumor foci was measured on formalin-fixed bread loaf slices imaged with the IVIS Spectrum and Lumina II imaging systems using the measurement tool in Living Image. The median fluorescence intensity (p/sec/cm2/sr) with interquartile range (IQR) was calculated per ROI from the fluorescent overlay image. If a single PTC focus was dissected in multiple bread loaf slices, average fluorescence intensity was calculated from ROIs drawn in each of the involved bread loaf slices. The average fluorescence intensity of healthy thyroid tissue was calculated from healthy thyroid tissue surrounding each PTC focus and averaged from multiple bread loaf slices if the associated PTC focus was dissected in multiple slices. The target-to-background ratio (TBR) was calculated by dividing the median fluorescence intensity (MFI) of the PTC focus by the MFI of adjacent healthy thyroid tissue. A PTC focus was defined as fluorescence-positive if a cutoff median fluorescence intensity was calculated at or above a threshold based on a receiver operator curve (ROC) for IVIS Spectrum. The receiver-operator curve (ROC) threshold was based on the optimal sensitivity for detecting PTC foci to maximize the detection of foci. A patient was diagnosed with multifocal disease using MFGI if at least two PTC foci with a fluorescence intensity above the threshold value were detected. Triple measurements of tumor and healthy thyroid tissues were taken using quantitative multidiameter single-fiber reflectance and single-fiber fluorescence (MDSFR/SFF) spectroscopy to adjust the optical attributes of the tissue. The intrinsic fluorescence value (Q.µafaxmm-1) from these measurements was calculated as described previously [20]. According to standard pathological procedure, all tissue was embedded in paraffin, assessed by a dedicated thyroid pathologist, and correlated to MFGI and spectroscopy data.

### Interim analysis

The purpose of the protocol was to determine the best dosage for detecting PTC nodal metastases. To achieve this, one or more cohorts were extended with three patients per cohort. This allowed for enough data points to be collected to select the dosage group with the optimal TBR for PTC nodal metastases. After the initial inclusion of nine patients, an interim safety analysis was carried out to evaluate the outcome measures and was reported to the Data Safety Monitoring Board. After the dose extension, a second interim examination was performed to ascertain the optimal dose. The cohort corresponding to this optimal dosage was subsequently expanded to encompass ten patients scheduled for surgical intervention for either primary or recurrent disease.

### Tracer binding validation

Two back-to-back 4-µm slides were acquired of formalin-fixed, paraffin-embedded (FFPE) blocks of all PTC foci and adjacent healthy thyroid tissue of the initial nine patients included in the dose-escalation study. The FFPE sections were used for H&E staining and assessment of EMI-137 fluorescence intensity. H&E staining was performed per standard of care and digitalized using a Hamamatsu flatbed scanner. An expert pathologist performed tissue segmentation. The Odyssey® CLX fluorescence flatbed scanning system (LI-COR Biosciences Inc., Lincoln, NE, USA) detected fluorescence in 4-µm-thick sections. All slides were scanned with the same imaging settings (wavelength 685 nm, resolution 21 µm, quality: highest, intensity: 5). Acquired fluorescence images were imported in ImageJ (Fiji, version 1.0) and segmented based on corresponding H&E slides. Mean fluorescence intensities (MFI) (arbitrary units) of PTC foci and adjacent healthy thyroid tissue were calculated. The TBR was calculated for each tumor focus by dividing the MFI of tumor tissue by the MFI of surrounding healthy thyroid tissue. To evaluate EMI-137 accumulation on a microscopic level in PTC and healthy material, a Leica SP8X DLS confocal microscope (Leica Biosystems GmbH, Wetzlar, Germany) was used for fluorescence microscopy on one representative 4-µm-thick slide per patient in the optimal dosage cohort. Consistent settings across magnifications on the I (DAPI, nuclei) and Y5 (Cy5, corresponding to EMI-137 excitation and emission) filter cube were used.

### Statistical analysis

Data with a normal distribution are depicted using mean values and range (min-max), while significance was determined using Student’s *t*-test. For data that were not normally distributed, they are displayed as a median accompanied by the interquartile range (IQR). To establish a device-specific threshold median fluorescent intensity for tumor foci in the ideal dosage group, receiver-operator curves (ROCs) were utilized. With this device-specific threshold, the diagnostic accuracy of MFGI for detecting tumor foci was calculated. A *p*-value < 0.05 was regarded as significant. Due to the limited size of some datasets, it was not feasible to determine range, IQR, or evaluate significance. All statistical analyses and graphic representations were carried out using GraphPad Prism (version 9.0, GraphPad Software Inc., San Diego, CA, USA).

## Results

### Patient characteristics

**Fig. 1:**
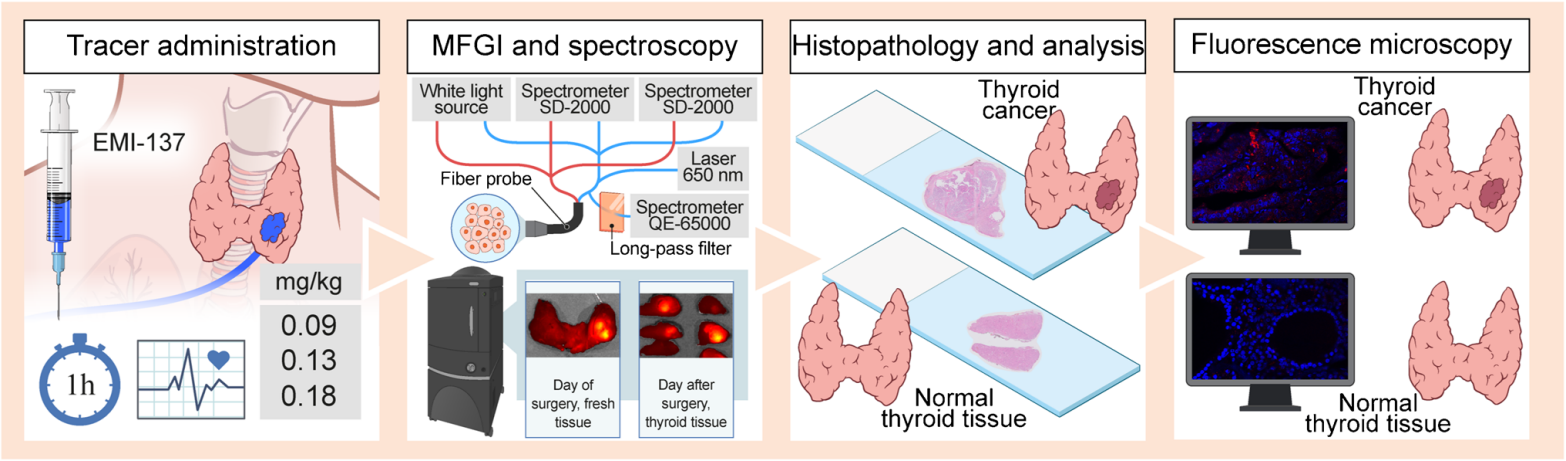
Study workflow illustrating the steps for tracer administration, imaging, analysis, and validation of in vivo tracer binding following intravenous administration. Abbreviations: EMI-137, investigational medicinal product; h, hour; mg, milligram; MFGI, molecular fluorescence–guided imaging; kg, kilogram

A total of 19 patients were included in the dose-escalation study. An overview of the study workflow is provided in Fig. 1. Patients undergoing lymph node dissection for recurrent disease were excluded from analysis (*n* = 5). Patients were treated with a hemithyroidectomy (*n* = 2) or total thyroidectomy (*n* = 12). Histopathological review confirmed PTC in all 14 patients who had (hemi)thyroidectomy, with multifocal disease in nine patients. All foci were confirmed as PTC. Patients underwent surgery at the University Medical Center Groningen (UMCG) (*n* = 13) or at the Erasmus University Medical Center (EMC) (*n* = 1); patient characteristics are shown in Table 1.

**Table 1:** Characteristics of patients included in the present study as selected from the lymph node metastases dose-finding study. Staging was performed according to the 8th edition of the American Joint Committee on Cancer staging system

	0.09 mg/kg (*n* = 1)	0.13 mg/kg (*n* = 8)	0.18 mg/kg (*n* = 5)	Total (*n* = 14)
*General characteristics*				
Female sex—*n* (%)	1 (100)	5 (62.5)	3 (60.0)	9 (64.3)
Male sex—*n* (%)	0	3 (37.5)	2 (40.0)	5 (35.7)
Age, years—median (IQR)	70.0 (-)	54.5 (26.5)	62.0 (34.5)	59.0 (26.8)
*Thyroid surgery*				
Hemithyroidectomy—*n* (%)	0	1 (12.5)	0	1 (7.1)
Total thyroidectomy—*n* (%)	1 (100)	7 (87.5)	5 (100)	13 (92.9)
*T-stage*				
pT1—*n* (%)	1 (100)	3 (37.5)	2 (40.0)	6 (42.8)
pT2—*n* (%)	0	0	3 (60.0)	3 (21.4)
pT3—*n* (%)	0	3 (37.5)	0	3 (21.4)
pT4—*n* (%)	0	2 (25.0)	0	2 (14.4)
*Multifocality on pre-operative ultrasound*				
Yes—*n* (%)	1 (100)	0	0	1 (7.1)
No—*n* (%)	0	8 (100)	5 (100)	13 (92.9)
*Multifocality* on histopathology				
Yes—*n* (%)	1 (100)	5 (62.5)	3 (60.0)	9 (64.4)
Bilateral—*n* (%)	1(100)	3 (37.5)	3 (60.0)	7 (50.0)
Unilateral—*n* (%)	0	2 (25.0)	0	2 (14.4)
No—*n* (%)	0	3 (37.5)	2 (40.0)	5 (35.6)
Foci analyzed by MFGI				
Primary PTC foci—*n*	1	8	5	14
Multifocal PTC foci—*n*	1	10	9	20
Foci analyzed by MDSFR/SFF spectroscopy				
Primary PTC foci—*n*	1	8	5	14
Multifocal PTC foci—*n*	0	6	2	8

*PTC* papillary thyroid cancer,* T* tumor stage

Patients received 0.09 mg/kg (*n* = 1), 0.13 mg/kg (*n* = 8), or 0.18 mg/kg (*n* = 5) EMI-137 intravenously. No serious adverse events were reported during the administration of EMI-137. However, one grade 1 adverse event could be associated with the use of EMI-137. In the 0.18 mg/kg dosage cohort, a patient experienced a self-resolving episode of flushing 25 min after the administration of EMI-137. Due to a technical malfunction with the IVIS Spectrum, three of the four patients from the 0.13 mg/kg dosage extension group, following the second interim analysis, were scanned using the IVIS Lumina II. After bread loaf slicing and correlation to histopathology, 34 primary PTC tumors (14 primary PTC foci and 20 multifocal PTC foci) were identified on final histopathology (Table 1).

### Visualization and quantification of EMI-137 fluorescence in tumor tissue

**Fig. 2:**
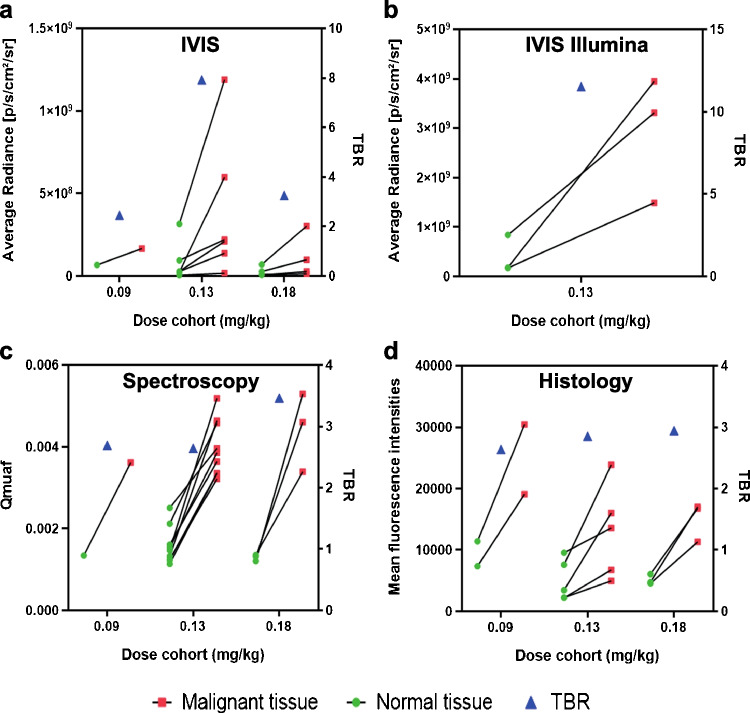
**a** An overview of fluorescent intensities per dosage cohort of formalin-fixed papillary thyroid cancer (PTC) and healthy thyroid tissue imaged with the IVIS Spectrum, the IVIS Lumina (**b**), and spectroscopy (**c**). The mean fluorescence intensity per dosage cohort acquired from histological slides is provided in **d**. Green dots resemble healthy thyroid tissue, red squares resemble malignant tissue, and blue triangles resemble the TBR per dosage cohort (depicted on the right *y*-axis)

**Fig. 3:**
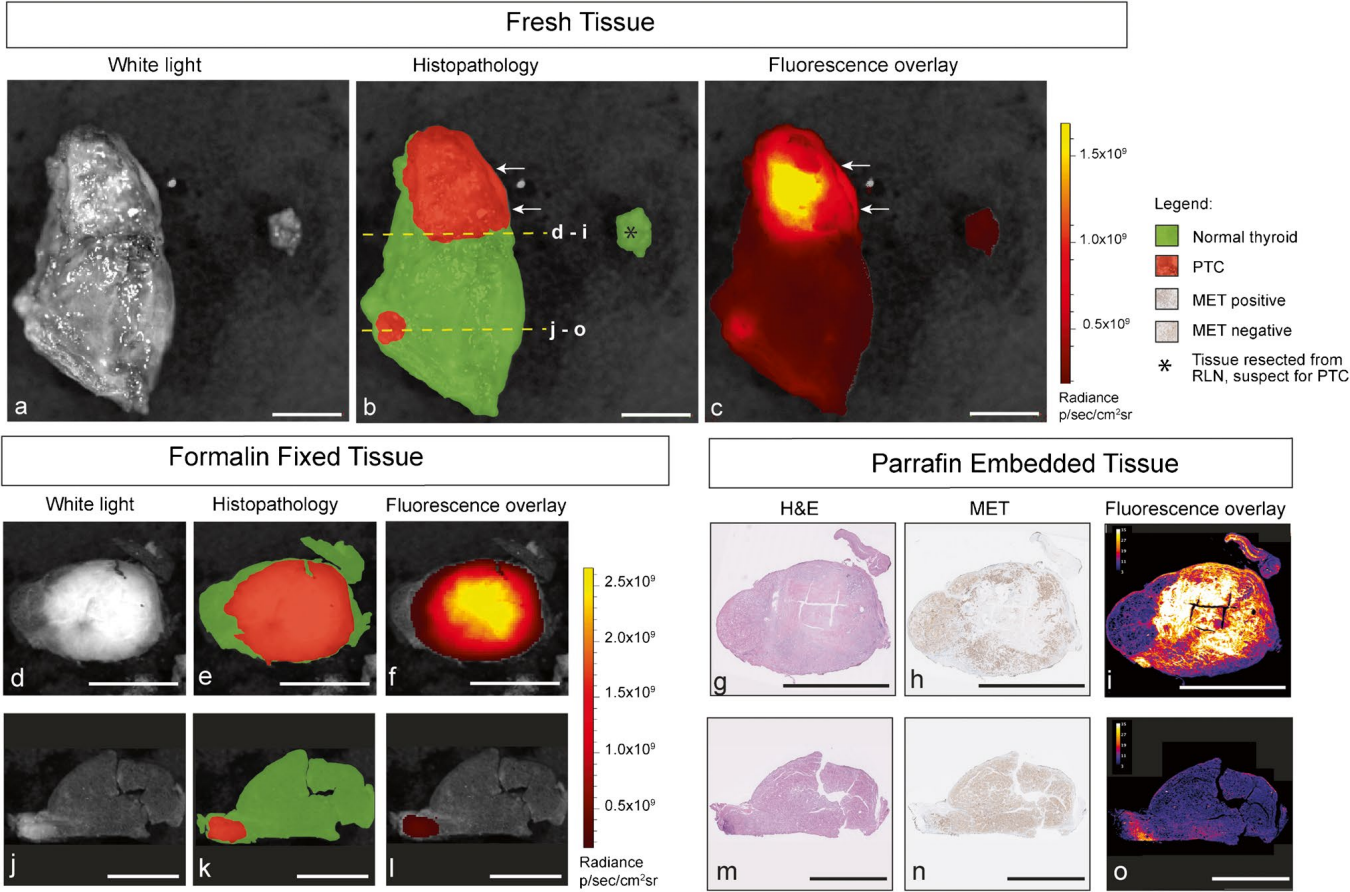
Representative images in the 0.13 mg/kg dosage cohort of a patient without detection of PTC multifocality on pre-operative ultrasound. The right thyroid lobe is imaged with the IVIS Spectrum as a fresh specimen from a dorsal perspective (**a**). The location of PTC foci was derived from the final histopathology results and drawn as a layer on the imaged fresh specimen (**b**). The primary tumor is in the cranial part of the right thyroid lobe. A second fluorescent focus located close to the ligament of Berry is visualized and confirmed as multifocal PTC on histopathological assessment (**m**–**o**). The asterisk marks a piece of tissue clinically suspect for PTC and resected from the recurrent laryngeal nerve. The fluorescence intensity of this tissue was comparable to healthy thyroid tissue (**b**, **c**). Histopathological assessment of the resected tissue from the RLN showed healthy thyroid tissue. The region between the two arrows indicates a region with a higher fluorescence signal outside the tumor, suggesting a tumor deposition (**b**, **c**). The histopathological assessment showed a positive resection margin. Dotted lines mark the locations of the bread loaf slices taken from the primary tumor (**d**–**f**) and secondary PTC focus (**j**–**l**), showing higher fluorescence intensity compared to surrounding healthy thyroid tissue. 4 mu tissue slices for H&E, anti-MET immunohistochemical staining, and fluorescent images were acquired from BLS of the primary tumor (**g**, **h**) and multifocal PTC (**m**–**o**). A higher MET staining (**h**, **n**) and fluorescence intensity (**i**, **o**) in PTC compared to adjacent normal tissue was confirmed. Fluorescence intensities are scaled and provided in radiance. Scale bars represent 10 mm. Abbreviations: PTC, papillary thyroid cancer; RLN, recurrent laryngeal nerve

To objectify the potential of EMI-137 to detect PTC foci, we first quantified fluorescence intensity of PTC foci and adjacent healthy thyroid tissue. Regardless of the imaging system (IVIS Spectrum or IVIS Lumina), we observed a higher fluorescence signal in PTC foci compared to adjacent healthy thyroid tissue in all dosage cohorts (Fig. 2a, b, Table 2). In the 0.13 dosage cohort, a TBR of 9.67 (range 2.30–26.27) was calculated, compared to 2.45 and 3.55 (range 1.39–4.27) in the 0.09 mg/kg and 0.18 mg/kg cohorts, respectively. Representative IVIS Spectrum images of multifocal PTC detected by MFGI and under-staged by pre-operative ultrasound are shown in Fig. 3.

**Table 2:** MFGI and MDSFR/SFF spectroscopy quantification values of 0.09 mg/kg, 0.13 mg/kg, and 0.18 mg/kg dosage cohorts

	0.09 mg/kg	0.13 mg/kg	0.18 mg/kg
*MFGI, p/sec/cm*^*2*^*/sr—median (IQR)*			
IVIS Spectrum			
PTC foci	16.6 × 10^7^	39.5 × 10^7^ (46.2 × 10^7^)	11.07 × 10^7^ (17.93 × 10^7^)
Adjacent healthy thyroid tissue	6.76 × 10^7^	8.27 × 10^7^ (7.22 × 10^7^)	2.92 × 10^7^ (4.10 × 10^7^)
IVIS Lumina			
PTC foci	n/a	29.1 × 10^7^	n/a
Adjacent healthy thyroid tissue	n/a	3.97 × 10^7^	n/a
*Spectroscopy, Q.µ*_*a*_^*f*^_*ax*_*mm*^*−1*^*—median (IQR)*			
Formalin fixed PTC foci	0.0036	0.0039 (0.0012)	0.0043
Formalin fixed adjacent healthy thyroid tissue	0.0013	0.0015 (0.0006)	0.0012

The median radiance (p/sec/cm^2^/sr) and median intrinsic fluorescence (Q.µ_a_^f^) calculated from MFGI and MDSFR/SFF spectroscopy, respectively, are provided for the dosage cohorts

*cm* centimeter, *IQR* interquartile range, *MDSFR/SFF* multidiameter single-fiber reflectance and single-fiber fluorescence, *MFGI* molecular fluorescence–guided imaging, *mm* millimeter, *n/a* not applicable, *p* photons, *PTC* papillary thyroid cancer, *sec* second, *sr* steradian, *kg* kilogram

To estimate the diagnostic accuracy of MFGI in the optimal dosage cohort, we determined a threshold for the median fluorescent intensities for the detection of PTC foci imaged with the IVIS Spectrum. We did not determine a device-specific threshold for the IVIS Lumina due to limited data. We determined 2.13 × 10^7^ p/sec/cm^2^/sr as the threshold for dosage cohort 0.13 mg/kg (supplementary Fig. 1), with a PTC foci-specific sensitivity of 66.66%, specificity of 33.33%, and positive predictive value of 50.0%. MFGI identified multifocal disease in four out of five patients (80.0%) diagnosed with histologically proven multifocal disease. In our high volume, high expertise thyroid cancer center, none of these patients was diagnosed with multifocal PTC by our expert radiologists by pre-operative ultrasound. The smallest PTC foci detected using MFGI was 1.40 mm in size.

### Quantification of intrinsic fluorescence values using MDSFR/SFF spectroscopy

Quantitative spectroscopy was performed in 13 patients to assess the intrinsic fluorescence values of PTC foci and corresponding adjacent healthy thyroid tissue (Table 1). One patient was excluded from the analysis due to a technical failure of the spectroscopy device. Compared to healthy thyroid tissue, PTC foci had higher intrinsic fluorescence values in the 0.13 mg/kg (*p* < 0.001) and 0.18 mg/kg (*p* < 0.001) dosage cohorts (Fig. 2c). A mean TBR of 2.50 (range 1.57–3.50) was detected in the 0.13 mg/kg cohort, compared to 2.69 (0.09 mg/kg cohort) and 3.47 (0.18 mg/kg cohort, range 2.50–4.39). An overview of the measured intrinsic fluorescence values for the PTC tumor foci per dosage cohort is provided in Table 2.

### Visualization of EMI-137 on a microscopic level in PTC foci

The fluorescence intensity of PTC foci and adjacent healthy thyroid tissue was acquired from 4-µm slices of nine PTC foci in five patients (dosage cohorts, 0.09 mg/kg *n* = 1; 0.13 mg/kg *n* = 2; and 0.18 mg/kg *n* = 2) and correlated to histopathology. Compared to healthy thyroid tissue, higher fluorescence intensity of PTC foci was found in the 0.09 mg/kg, 0.13 mg/kg (*p* = 0.04), and 0.18 mg/kg (*p* = 0.02) dosage cohorts (Fig. 2d). A mean TBR of 2.85 (range 1.4–4.6) was detected in the 0.13 mg/kg cohort, compared to 2.60 (0.09 mg/kg cohort) and 2.90 (0.18 mg/kg cohort, range 2.49–3.57). Representative images are shown in Fig. 3g–i and m–o.

### Fluorescence microscopy and the expression of MET

**Fig. 4:**
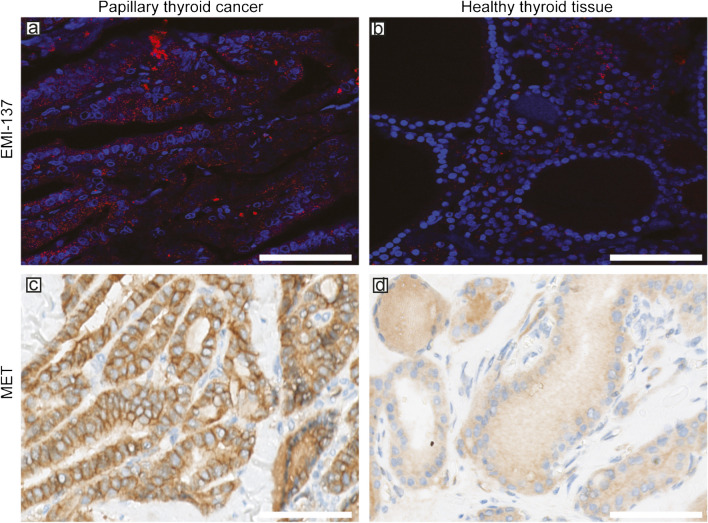
Fluorescence microscopy of papillary thyroid cancer (**a**) and healthy thyroid tissue (**b**) of patients who underwent EMI-137 administration with nuclei (blue) and EMI-137 (red). To assess MET expression status, immunohistochemical staining using 8191S was performed on a 4-µm slice acquired from the same PTC (**c**) and healthy thyroid tissue (**d**) as used for fluorescence microscopy. The scale bar represents 75 µm

Cross-sectional confocal fluorescent microscopy imaging confirmed a clear cytoplasmic EMI-137 signal in PTC foci. In contrast, the observed cytoplasmic concentration of EMI-137 in healthy thyroid tissue was significantly lower. A positive MET staining status was predominantly observed in PTC foci, while it was nearly nonexistent in healthy thyroid tissue (Fig. 4a–d).

### Additional potential clinical value of MFGI with EMI-137 in patients with PTC

During surgery, a severe complication is an injury to the recurrent laryngeal nerve. In two patients, the surgeon resected tissue clinically suspicious for PTC from the nerve with the possible risk of causing recurrent laryngeal nerve injury and potential paralysis, either temporary or permanent. After the histopathological assessment, the resected tissue in both patients was tumor negative, and fluorescence intensity was similar to healthy thyroid tissue. Figure 3a–c shows representative fluorescent images of the resected tissue. In one patient, MFGI was able to detect a histologically proven positive resection margin (Fig. 3a–c). Using MFGI as a tool may thus help the surgeon to prevent the resection of benign tissue lowering the risk of injury or complications.

## Discussion

This study showed the detection and quantification of PTC using MFGI after intravenously administering EMI-137 prior to surgery. Using MFGI, we were able to detect multifocal disease missed by pre-operative ultrasound, potentially intra-operatively upstaging patients. We found a higher fluorescent signal in PTC foci compared to adjacent healthy thyroid tissue after assessment with MFGI and spectroscopy. MFGI showed a mean TBR of 9.67 in dosage cohort 0.13 mg/kg. On a microscopic level, both MET expression status and mean fluorescence intensity of PTC foci were higher compared to healthy thyroid tissue, suggesting selective binding of EMI-137 in PTC foci.

MET overexpression in primary PTC and nodal metastases was previously confirmed at both the gene and protein levels compared to healthy thyroid and nodal tissue [12, 15–19, 21–29]. A recent study concluded that MET-targeted MFGI and spectroscopy of PTC nodal metastases is feasible and safe and might decrease the number of negative central compartment dissections [12]. A dosage of 0.13 mg/kg EMI-137 was identified to be optimal for the detection of PTC nodal metastases. Another advantage of EMI-137 is the injection-imaging interval between 1 and 3 h [30] compared to other clinically available tracers requiring administration up to multiple days prior to surgery [31–33]. This greatly enhances the peri-operative applicability of EMI-137.

The reliability of the acquired intra-operative fluorescent signal by either MFGI or spectroscopy depends on the specific binding of EMI-137 to PTC foci. EMI-137 was previously shown to accumulate in thyroid cancer cell lines and PTC nodal metastases, whereas the cytoplasmatic localization in normal lymph nodes was nearly absent [12]. Our study adds that EMI-137 also agglomerates in primary PTC foci in vivo but is nearly absent in healthy thyroid tissue. This further supports the hypothesis that circulating EMI-137 binds specifically to MET-expressing PTC tumor cells.

Important strengths of our study are the multicenter setup and application of a multi-step analytical framework to demonstrate PTC tumor-specific targeting of EMI-137 on both macroscopic and microscopic level. Moreover, the smallest malignant tumor focus that was detectable with IVIS imaging measured 1.40 mm in diameter, thereby showing the potential diagnostic accuracy of this near-infrared fluorescent tracer. Using MFGI, we detected multiple PTC foci in patients pre-operatively diagnosed with unifocal disease by expert radiologists resulting in the possibility of upstaging these patients to a high-risk tumor. The PTC foci-specific positive predictive value of MFGI, 50%, is within the reported range of the positive predictive value (PPV) of ultrasound for detecting multifocal disease, 44.4–57%. However, the MFGI PPV was calculated in a cohort of patients with a negative pre-operative ultrasound. Therefore, the real-world PPV of the detection of multifocality by MFGI is expected to be higher than the gold standard of pre-operative ultrasound staging [10, 34].

Our study has several limitations. First, the study was not primarily designed to select the optimal dosage of EMI-137 for the detection of primary PTC. The patients were earlier included in a dose-escalation study with the identification of PTC nodal metastases as primary endpoint. Secondly, the emission wavelength of 675 nm for EMI-137 might offer a more superficial penetration depth in comparison to alternative targeted tracers. This may lead to interference with background autofluorescence during intra-operative imaging.

Future studies should investigate the intra-operative assessment of the thyroid for the presence of a fluorescent signal after administration of a targeted NIRF tracer, indicating tumor foci. A phase-2 study will focus on diagnostic accuracy for the detection of primary PTC and multifocal disease and its added value compared to ultrasound staging. MFGI could be a valuable tool for intra-operatively restaging patients to a high-risk tumor, thus benefitting from current extensive treatment and not performing the de-escalated treatment based on pre-operative ultrasound, as the 2015 ATA guidelines proposed [1]. This phase-2 study can be used to investigate the diagnostic accuracy of the detection of PTC positive margins, possibly reducing morbidity [36, 37] and for a more selective lymph node dissection for nodal metastases. This study may also investigate treatment-related morbidities such as parathyroid injury and laryngeal nerve injury impacting the quality of life [3–5]. Results from our study could complement other pre-operative imaging modalities, such as multispectral optoacoustic tomography (MSOT), paving the way to targeted NIRF-tracer enhanced MSOT of the thyroid and nodal compartments. Handheld optoacoustic imaging can visualize the thyroid, malignant and benign thyroid nodules, and surrounding structures in vivo [35–37]. Future studies are needed to assess the feasibility of NIRF-tracer enhanced MSOT of the thyroid, nodal compartments, and its benefits compared to the current gold standard for pre-operative staging by ultrasound.

In conclusion, MET-targeted MFGI and spectroscopy of primary PTC using EMI-137 are a very promising modality to identify multifocal disease. PTC tumor-specific targeting of EMI-137 was confirmed on both macroscopic and microscopic level. MFGI and spectroscopy could be useful in identifying multifocal disease, ultimately contributing to intra-operative upstaging and subsequent total thyroidectomy.

### Supplementary Information

Below is the link to the electronic supplementary material.

Supplementary file1 (DOCX 51 KB)

## Data Availability

The study protocol and deidentified data used for this study are available upon request via the corresponding author after approval of all authors, review of a proposal, and following the establishment of data transfer agreements.
