# Supplementary material for: MET-receptor targeted fluorescent imaging and spectroscopy to detect multifocal papillary thyroid cancer
Source: Eur J Nucl Med Mol Imaging. 2023 Nov 29;51(8):2384–94. doi: 10.1007/s00259-023-06525-5 (PMC11178647; doi:10.1007/s00259-023-06525-5)
Supplement: Supplementary file 1 — Supplementary file1 (DOCX 51 KB) [file 259_2023_6525_MOESM1_ESM.docx]

**Supplemental figures**

***Supplemental figure 1.*** ***Sensitivity and specificity of near-infrared fluorescent tracer EMI-137.*** *The figure shows the receiver operator curve for 15 PTC foci, including the 15 adjacent healthy thyroid tissue in 5 patients in the 0.13 mg/kg dosage cohort and imaged with the IVIS spectrum.*
